# Simulation and evaluation of freeze-thaw cryoablation scenarios for the treatment of cardiac arrhythmias

**DOI:** 10.1186/s12938-015-0005-9

**Published:** 2015-02-18

**Authors:** Michael Handler, Gerald Fischer, Michael Seger, Roland Kienast, Friedrich Hanser, Christian Baumgartner

**Affiliations:** Institute of Electrical and Biomedical Engineering, University for Health Sciences, Medical Informatics and Technology, Eduard Wallnöfer-Zentrum 1, Hall in Tirol, 6060 Austria; AFreeze GmbH, Eduard Bodem Gasse 8, Innsbruck, 6020 Austria; Medical Engineering and Healthcare IT, Carinthia University of Applied Sciences, Primoschgasse 10, Klagenfurt, 9020 Austria; Institute of Health Care Engineering with European Notified Body of Medical Devices, Graz University of Technology, Kopernikusgasse 24, Graz, 8010 Austria

**Keywords:** Cardiac cryoablation, Modeling and simulation, Ablation scenarios, Freeze-thaw cycles, Transmural temperature distribution, Cooling rates, Thawing rates, Cryoadhesion

## Abstract

**Background:**

Cardiac cryoablation is a minimally invasive procedure to treat cardiac arrhythmias by cooling cardiac tissues responsible for the cardiac arrhythmia to freezing temperatures. Although cardiac cryoablation offers a gentler treatment than radiofrequency ablation, longer interventions and higher recurrence rates reduce the clinical acceptance of this technique. Computer models of ablation scenarios allow for a closer examination of temperature distributions in the myocardium and evaluation of specific effects of applied freeze-thaw protocols in a controlled environment.

**Methods:**

In this work multiple intervention scenarios with two freeze-thaw cycles were simulated with varying durations and starting times of the interim thawing phase using a finite element model verified by in-vivo measurements and data from literature. To evaluate the effects of different protocols, transmural temperature distributions and iceball dimensions were compared over time. Cryoadhesion durations of the applicator were estimated in the interim thawing phase with varying thawing phase starting times. In addition, the increase of cooling rates was compared between the freezing phases, and the thawing rates of interim thawing phases were analyzed over transmural depth.

**Results:**

It could be shown that the increase of cooling rate, the regions undergoing additional phase changes and depths of selected temperatures depend on the chosen ablation protocol. Only small differences of the estimated cryoadhesion duration were found for ablation scenarios with interim thawing phase start after 90 s freezing.

**Conclusions:**

By the presented model a quantification of effects responsible for cell death is possible, allowing for the analysis and optimization of cryoablation scenarios which contribute to a higher clinical acceptance of cardiac cryoablation.

## Background

Cardiac cryoablation (CCA) is a minimally invasive therapeutic procedure using cryocatheters to treat cardiac arrhythmias by cooling cardiac tissues responsible for the cardiac arrhythmia to freezing temperatures. Compared to the alternative radiofrequency ablation (RFA), in which the target tissue is heated, the use of refrigerant offers some advantages, e.g. less endothelial disruption and the capability of the applicator to remain at the target location during the ablation due to cryoadhesion [1-3]. Compared to RFA, longer intervention durations and higher recurrence rates were reported for CCA [2,4,5]. In order to improve applied cryoablation protocols and, consequently, the outcome of CCA, a better knowledge about the temperature progress in the treated myocardium during the procedure is necessary. Multiple studies are available investigating the effects and temperature distributions/profiles during CCA in-vivo (a list of in-vivo studies can be found in [6]), in-vitro (e.g. [7,8]) and in-silico [9-11].

In-vivo and in-vitro studies showed that cardiac tissue is sensitive to freezing injury [6,8,12]. Weimar et al. [8] used -20°C as lethal boundary for myocardial cells in their study, which is a relatively warm lethal temperature compared to other types of tissue (e.g. -50°C is proposed as lethal temperature for tumor cells [6]). A lethal temperature range between -10°C and -25°C was also confirmed when applied for longer durations [3,6]. Additional parameters for acute and delayed cell death were identified. A detailed description of ablation parameters, such as cooling rate, tissue temperature, duration of freezing, thawing rate, the repetition of the freeze-thaw cycle and the interval between freeze-thaw cycles can be found in [6] and [12].

A reason for the enhanced cell death by repeated freeze-thaw cycles is the cooling rate increase at the beginning of the second freezing phase [6] caused by precooled surrounding tissue and a reduced heat contribution of tissue ablated during the first freeze-thaw cycle. This cooling rate enhancement consequently leads to more extensive intracellular ice crystallization, which is one of the main indicators for acute cell death caused by disruption of cell membranes and organelles [13].

Another factor increasing cell death by multiple freeze-thaw cycles is ice recrystallization and increased solute effects primarily caused by slow thawing rates over longer durations. For longer thawing phases microcirculation fails and the heat contribution in this area lessens, which allows for a more effective second freezing phase [6].

The ablation parameters of (multiple) freeze-thaw cycles are strongly dependent on the distance of the tissue to the applicator: During the first freezing phase cell death caused by intracellular ice crystallization mainly occurs in areas close to the applicator, while cell death by solute effects is primarily seen in areas farther away from the cryoprobe [12]. Multiple freeze-thaw cycles can be used to extend these effects into areas more distant from the cryoprobe [6,12]. To analyze the aforementioned effects in the myocardial tissue during different cryoablation scenarios, a detailed examination of the temperature distribution over time is necessary.

Wood et al. compared lesion dimensions and measured tissue temperatures during cryoablation in-vitro using tip applicators with different dimensions, applicator orientations, contact pressures and the presence/absence of superfusate flow [7]. The analysis of lesion dimensions showed that all described factors have an influence on the final ablation outcome. The effects leading to an increase of lesion dimensions during the performed single freeze-thaw cycle (i.e. higher cooling rates and lower minimal temperatures) are also reflected by the recorded temperature profiles in the myocardium.

Proper mathematical models allow for analyzing temperature distributions during cryoablation procedures without biases introduced by anatomical variations or the experimental setup. In a work of Chua and Chou, a computer model was created to study the thermal processes during cryoablation in porcine liver tissue [14]. In the simulations of this study, the impact of different thawing durations, thawing temperatures and number of freeze-thaw cycles on the ablated tissue was compared in terms of calculated ice volumes and cell survival signatures based on varying cooling and thawing rates. The study showed that longer thawing periods, passive thawing and the repetition of freeze-thaw cycles positively affect the ablation outcome. The simulations demonstrated that the ablation effect on the tissue also depends on the distance to the cryoapplicator surface.

Only a few studies exist focusing on the simulation of temperature distributions/profiles and corresponding effects on lesion dimensions for cardiac cryoablation. In a previous study by Seger et al. myocardial temperature distributions were simulated for different freezing outlet configurations in a loop shaped cryocatheter [9]. In this study lesion dimensions were estimated based on the simulated ice formation after 300 s freezing. It was found that the configuration of the freezing outlets significantly influences the transmural temperature distribution and, consequently, the lesion characteristics. Findings obtained by these simulations could be confirmed by morphological and histological examinations.

Recently a computer model based on Pennes’ bioheat equation [15,16] was developed by our group simulating the temperature distribution in a myocardial tissue layer with 6 mm thickness and a blood layer in contact with the applicator and the endocardium [10]. This model takes into account the different phases of the refrigerant flow in the applicator in order to consider these effects in the temperature distribution of the simulated tissue layer. Ice formation (phase change from liquid to solid) influences the thermal properties of the tissue (specific heat capacity, latent heat, thermal conductivity, metabolic heat generation and blood perfusion) and the blood layer (material properties as described for tissue without metabolic heat generation), and was integrated into the model by the effective heat capacity model [16]. The model was verified by in-vivo measurements at the applicator tip and lesion dimensions from literature. Therefore it constitutes a good basis for the investigation of transmural temperature profiles and distributions during different freeze-thaw scenarios, which is the main focus of this work.

In this work effects on transmural temperature distributions, temperature profiles and ice dimensions of different thawing durations and thawing start times were analyzed using a slightly adapted variant of our previously presented model [10]. Characteristic parameters of the different phases were quantified over transmural depth and evaluated for various ablation scenarios. Furthermore, the temperatures at the applicator surface being in direct contact with the endocardium were selected during the thawing phases to estimate the length of time until the applicator loses cryoadhesion and disengages from the target ablation area.

## Methods

### Model

**Figure 1:**
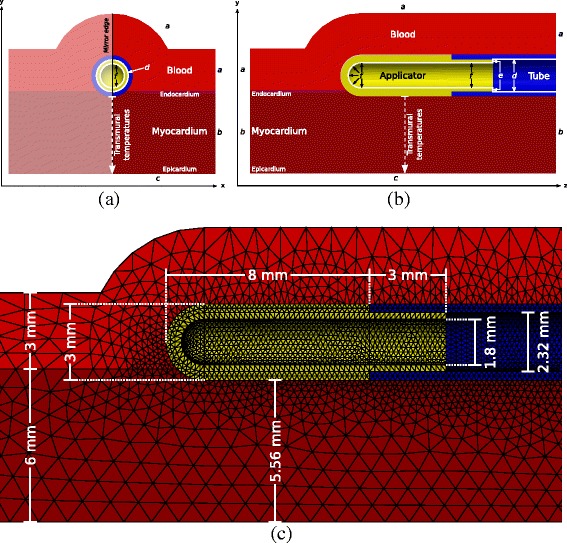
**Model used for the simulation of temperature fields.** Segment of model used for the simulation of temperature fields during CCA similar to the model presented in [10]. **(a)** Cross section of applicator’s short axis. **(b)** Cross section of applicator’s long axis. **(c)** FEM meshing and geometry dimensions.

To simulate the temperature distribution in the myocardial tissue and the components of the ablation system, a true to scale model of a 9 Fr 8 mm applicator (Freezor MAX, Medtronic Inc., Minneapolis, MN) applied to myocardial tissue with 6 mm transmural thickness was used, a slightly modified variant of the model presented in [10] (see Figure 1). The segment of the tip applicator not attached to the endocardium is surrounded by a 3 mm blood layer to simulate the blood stream by highly perfused elements [10]. A thicker blood layer was simulated in this study allowing to consider temperatures more distant from the applicator. Additionally, the tissue area in contact with the applicator was reduced to conform to applicator contact areas from literature [17]. The described model was prepared with the software HyperWorks 12 (Altair Eng. Inc.) and simulated using the Finite Element Method (FEM). The meshed geometry comprises of 39160 vertices and 214526 tetrahedrons. As temporal differencing scheme the Crank-Nicolson method [18] was used with a temporal resolution of 10 ms.

To estimate the temperature distribution in living myocardial tissue during the simulated ablation protocols *Pennes’ bioheat equation* was used [10,15,16]:

(1) $$\begin{array}{@{}rcl@{}} \rho C(\mathbf{X},T)\frac{\partial T(\mathbf{X}, t)}{\partial t} = \nabla \cdot \lambda(\mathbf{X},T) \nabla T(\mathbf{X}, t) + Q_{p}(\mathbf{X}, T) + Q_{m}(\mathbf{X}, T), \quad \mathbf{X} \in \Omega \end{array} $$

*ρ*, *C* and *λ* are the material and temperature dependent density (kg m ^−3^), specific heat capacity (J kg ^−1^ °C ^−1^) and thermal conductivity (W m ^−1^ °C ^−1^) at location **X**, time *t* (s) and temperature *T* (°C) in the modeled spatial domain *Ω*. To model the thermal load of blood perfusion and metabolic heat generation a blood perfusion term *Q*_*p*_ (W m ^−3^) and a metabolic heat contribution term *Q*_*m*_ (W m ^−3^) were integrated into the model. The fusion enthalpy of freezing blood and tissue was applied proportionally to the phase transition range between -10°C and 0°C (effective heat capacity model [16]) [10,19].

To incorporate the heat contribution of adjacent regions in Figure 1 (boundaries *a*, *b* and *c*) *Cauchy boundary conditions* were applied. The cooling flux of the refrigerant (boundaries *d*, *e* and *f*) was modeled by time and temperature dependent *Neumann boundary conditions* and *Cauchy boundary conditions* to incorporate different phases of the refrigerant during a freeze-thaw cycle.

For a detailed description of the perfusion term and metabolic heat contribution term, distinct material properties and applied boundary conditions used in the model, we refer to [10]. Due to the experimental model validation carried out in our previous work [10], the boundary condition of the epicardial surface *c* was set to approximate open chest conditions. For this study we slightly adapted this boundary condition to the closed chest situation using the values of Seger et al. [9] by increasing the heat transfer coefficient *α* to 200 W m ^−2^ °C ^−1^ and the external temperature *T*_*c*_ to body temperature (36.5°C). The simulated temperature fields were compared with our previous model [10] showing a similar characteristic at the applicator tip. However, reduced ice volumes in the tissue were detected, which are primarily caused by the increased heat transfer coefficient at the epicardial boundary.

### Ablation scenario evaluation

**Figure 2:**
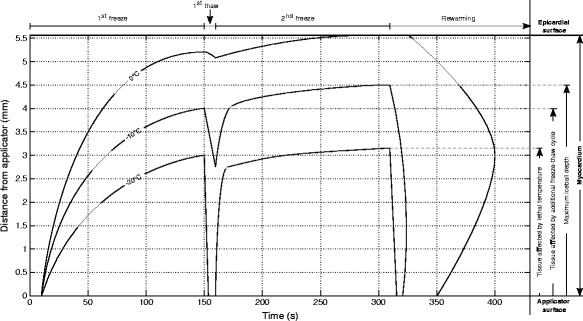
**Isotherms of schematic transmural temperature depths over time.** Isotherms of schematic transmural temperature depths over time for a cryoablation scenario with two freeze-thaw cycles (150 s freezing followed by 10 s thawing, 150 s freezing and rewarming).

To investigate the relevant ablation measures (minimal temperatures, cooling and thawing rates, progress of phase change boundaries) of different scenarios with two freeze-thaw cycles, transmural temperature profiles were computed between the coolest point at the epicardium and the applicator, and selected isotherms were extracted (see schematic overview of transmural temperature profiles in Figure 2). Furthermore, the ice volume (tissue below solidus temperature of -10°C) was calculated and compared between different protocols.

**Figure 3:**
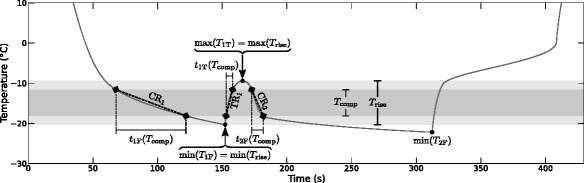
**Temperature and time values/ranges used for comparable cooling/thawing rate computation.** Schematic temperature profile in myocardium with characteristic temperature values, and temperature and time ranges for the calculation of comparable cooling rates for the first and second freezing phase (CR_1_, CR_2_) and thawing rates for the interim thawing phase (TR_1_). *T*_rise_ is the temperature range during the first thawing phase, min(*T*_1F_) and min(*T*_2F_) define the minimal temperatures of the first and second freezing phase, respectively, max(*T*_1T_) is the maximal temperature during the interim thawing phase, and *t*_1F_(*T*_comp_), *t*_2F_(*T*_comp_) and *t*_1T_(*T*_comp_) are the durations needed to pass the comparative temperature range *T*_comp_ in the first and second freezing phase and the interim thawing phase, respectively.

To evaluate the influence on cooling rates in different transmural depths average cooling rates were calculated based on transmural temperature profiles. The average cooling rate CR_*i*_ (° C s^−1^) of freezing phase *i* (first freeze *i*=1, second freeze *i*=2) was calculated by

(2) $$ \text{CR}_{i} = \frac{T_{\text{comp}}}{t_{i\mathrm{F}}(T_{\text{comp}})}\text{,}   $$

where the temperature interval *T*_comp_ (°C) (dark gray temperature range in Figure 3) is a central part of the temperature range during the first thawing phase *T*_rise_ (°C) (light gray temperature range in Figure 3). *T*_rise_ is defined as the temperature interval between the minimal temperature during the first freezing phase (min(*T*_1F_) (°C) in Figure 3) and the maximal temperature during the interim thawing phase (max(*T*_1T_) (°C) in Figure 3). Due to the slow temperature reduction at the beginning of the second freezing phase (see temperature curve progression right after max(*T*_1T_) in Figure 3), only the central temperature range covering 60% of *T*_rise_ was used for the cooling rate computation of the first and second freezing phase in this study. *t*_*i*F_(*T*_comp_) (s) defines the time needed to pass the temperature interval *T*_comp_ in freezing phase *i*.

The thawing rate for the simulated protocols was calculated correspondingly using the time range *t*_1T_(*T*_comp_) needed to pass *T*_comp_ in the interim thawing phase in the denominator of Equation 2.

### Simulated ablation scenarios

**Figure 4:**
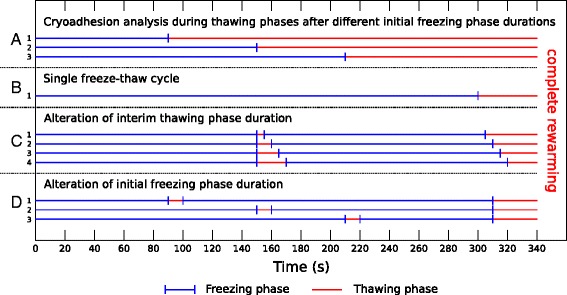
**Schematic overview of simulated ablation scenarios.** **A**: Cryoadhesion analysis during thawing phases after initial freezing phase durations of 90 s (A1), 150 s (A2) and 210 s (A3). **B**: Single freeze-thaw cycle with 300 s freezing and final rewarming (B1). **C**: Alteration of interim thawing phase duration (C1: 5 s, C2: 10 s, C3: 15 s, C4: 20 s) after 150 s freezing followed by a second freezing phase of 150 s. **D**: Alteration of initial freezing phase duration (D1: 90 s, D2: 150 s, D3: 210 s) followed by an interim thawing phase of 10 s in scenarios with two freeze-thaw cycles with a cumulated freezing duration of 300 s.

One of the main factors influencing the lesion dimension and destruction rate in multiple freeze-thaw cycles is the thawing phase between two freezing phases. In a first step the cryoadhesion duration of the applicator was analyzed during the thawing phase after previous freezing phases with different durations (see schematic ablation scenarios A1-A3 in Figure 4). To specifically investigate the effect of different thawing scenarios with two freeze-thaw cycles the accumulated freezing time was set to 300 s and the thawing duration and thawing start (initial freezing duration) were altered in the simulations (see schematic ablation scenarios B-D in Figure 4).

The duration of thawing between two consecutive freezing phases is one of the main factors affecting the efficiency of multiple freeze-thaw cycles. While longer thawing periods lead to increased efficacy due to recrystallization and osmotic damage [6,12], the adhesion of the applicator to the treated myocardium is negatively affected through longer thawing phases. The applicator remains attached to the endocardium by ice formation around the applicator until the tissue region in contact with the applicator undergoes phase transition from solid to liquid. To evaluate the thawing duration with preserved adhesion, the average temperature of the endocardial surface in contact with the applicator tip was analyzed in the thawing phase after initial freezing of 90 s, 150 s and 210 s. Once the average temperature of the endocardial tissue reaches the solid phase transition temperature of -10°C the applicator starts to disengage from the myocardial tissue. It should be noted that this does not imply an immediate loss of adhesion of the applicator after this short thawing period. The phase change from solid to liquid is not completed until the previously subtracted latent heat is added to the tissue again. The target temperature of -10°C was chosen to consider the “worst case” in our analysis. In our simulations the duration of the interim thawing phase after 150 s freezing was varied in a small range around the estimated minimal thawing time with persisting cryoadhesion. Based on the simulation results transmural temperature profiles were extracted to analyze effects, which influence the ablation outcome.

The tissue volume affected by repeated freezing is influenced in a high degree by the duration of the first freezing phase due to recrystallization of ice crystals developed during the initial freezing of tissue. To analyze temperature profiles of involved tissue regions for variable lengths of initial freezing phases, the first thawing phase of 10 s was started between 90 s and 210 s after the beginning of the first freezing phase. The duration of the second freezing phase corresponds to the time remaining to 300 s freezing. Analogous to the measurements for different thawing durations transmural temperature profiles, ice volumes and the increase of cooling rates were extracted over time and compared for different starting times of the interim thawing phase.

## Results

To compare the influence of different freeze-thaw cycle variants on the ablated myocardial tissue, cryoadhesion durations were estimated, and transmural temperatures and ice volumes (myocardial tissue volume with temperatures lower than the solidus temperature of -10°C) were extracted from the simulated temperature fields. In addition, cooling and thawing rates between the succeeding freezing phases of the simulated variants were compared.

### Cryoadhesion analysis

To estimate the thawing duration without losing the effect of cryoadhesion a deactivation of the applicator was simulated after 90 s, 150 s and 210 s of freezing and the time was measured until the contact area between applicator and myocardium reaches an average temperature of -10°C. Mainly due to the heating effect of the surrounding blood stream this temperature boundary is already reached after approximately 12 s (11.7 s) thawing in the scenario with 150 s initial freezing phase (scenario A2 in Figure 4). After this time the contact area between applicator and tissue reaches the lower boundary of the phase change temperature range in the simulation. The liquid phase change boundary of 0°C is reached at the surface between applicator and tissue after deactivating the applicator for approximately 40 s (41.5 s). The thawing duration until the applicator surface in contact with the tissue reaches the lower phase change boundary is similar compared to the investigated protocols with differing thawing starting times. When the applicator is deactivated after 90 s freezing (scenario A1 in Figure 4), the boundary between the applicator and the myocardial tissue reaches an average temperature of -10°C after 11 s and an average temperature of 0°C after 40.2 s. For the longer initial freezing phase of 210 s (scenario A3 in Figure 4) the thawing duration increases slightly to 12 s until a mean temperature of -10°C is reached at the applicator/tissue boundary. After 41.9 s the boundary between applicator and tissue warms up to 0°C.

### Single freeze-thaw cycle

**Figure 5:**
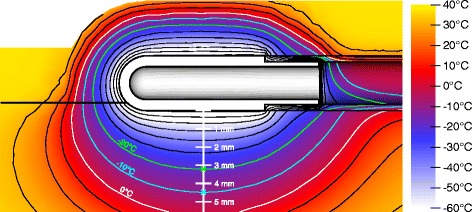
**Simulated temperature distribution after 300 s freezing.** Black isotherms denote 10°C steps. 0°C, -10°C and -20°C isotherms are highlighted in white, cyan and green, respectively. Crosses at -10°C and -20°C isotherms depict the maximal depth of the corresponding isotherm.

**Figure 6:**
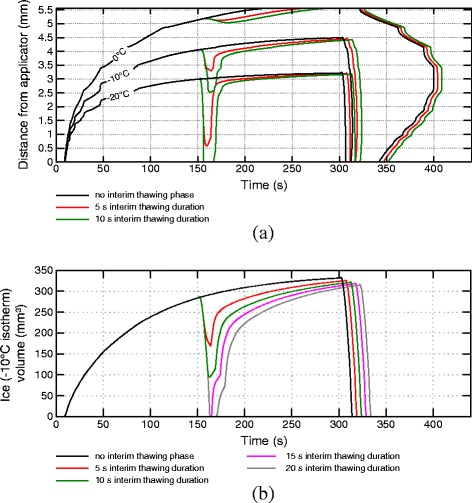
**Isotherms of transmural temperatures and ice volumes for varying interim thawing phase durations.** Isotherms of transmural temperatures **(a)** and ice volumes **(b)** over time for ablation protocols with 150 s initial freezing phase, interim thawing phases with varying duration, 150 s second freezing phase and final rewarming phase.

To obtain a reference of transmural temperature distribution and ice volume growth over time a single freeze-thaw cycle of 300 s freezing followed by rewarming was simulated (scenario B1 in Figure 4). Figure 5 depicts the temperature distribution at the end of the freezing phase. After 300 s freezing, the frozen volume reaches a transmural depth of 4.5 mm relative to the applicator surface, and the myocardium within a transmural distance of 3.2 mm is frozen below the necrotic temperature of -20°C (see Figure 6a and Table 1). The transmural extent of the iceball is comparable with lesion depths measured in an in-vivo study of Khairy et al. [20] (4.8 ± 2.7 mm) using a 9 Fr 8 mm applicator for CCA in adult miniature swine. Tse et al. [17] obtained similar lesion depths in a thigh muscle preparation to simulate intracardiac conditions during cryoablation using a 10 Fr 6.5 mm tip applicator with similar tissue contact area (4.9 ± 1.5 mm).

**Table 1:** **Transmural isotherm depths and ice volumes for varying interim thawing phase durations**

**Thaw dur.**	**1F**			**1T**			**2F**		
**(s)**	**-10°C**	**-20°C**	**IV**	**-10°C**	**-20°C**	**IV**	**-10°C**	**-20°C**	**IV**
	**(mm)**	**(mm)**	**(mm** ^**3**^ **)**	**(mm)**	**(mm)**	**(mm** ^**3**^ **)**	**(mm)**	**(mm)**	**(mm** ^**3**^ **)**
0	X	X	X	X	X	X	4.5	3.2	333
5	4.1	3.0	286	3.3	0.6	170	4.4	3.2	326
10	⋮	⋮	⋮	2.5	0.0	94	4.4	3.2	322
15	⋮	⋮	⋮	0.0	0.0	0	4.4	3.2	319
20	4.1	3.0	286	0.0	0.0	0	4.4	3.2	316

Transmural depths of selected isotherms and ice (= tissue below solidus temperature of -10°C) volumes (IV) for ablation protocols with an initial freezing phase of 150 s, an interim thawing duration between 0 s and 20 s, a second freezing phase of 150 s and final rewarming.

A maximal frozen volume of 333 mm ^3^ is reached at the end of the freezing phase, which is the simulated maximum iceball volume for all simulated scenarios (see Figure 6b and Table 1).

### Thawing duration

Figure 6 shows the transmural temperature and ice volume progress for different thawing durations after an initial freezing phase of 150 s (scenarios C1-C4 in Figure 4). In Figure 6a, isotherms of the transmural temperature distribution over time are plotted for the protocols without interim thawing phase and interim thawing phases of 5 s and 10 s after 150 s initial freezing, followed by a second freezing phase of 150 s and final rewarming. While tissue within 2.3 mm of transmural distance reaches the necrotic temperature of -20°C within the first minute of freezing, the myocardium more distant from the applicator shows a comparably slow decrease of temperature indicated by the slow increase of isotherms. The transmural extent of the iceball after 150 s simulation (4.1 mm) is comparable with lesion depths described in the study of Tse et al. [17] after 150 s freezing (3.8 ± 1.0 mm). Once the first freezing period ends after 150 s, the distances of the depicted isotherms to the applicator reduce rapidly due to the strong thermal load of the blood stream. In contrast, only a marginal reduction of the 0°C isotherm can be seen. The slow rewarming of areas with temperatures above the solidus temperature (-10°C) is caused by the latent heat required for the phase change, which must be added to the tissue again before a further rise in temperature is possible. During the rewarming of tissue an increase of solute effects and recrystallization of previously developed ice crystals takes place. The impact of these effects is stronger the longer the thawing duration lasts [6,12]. Additionally, the transmural zone entering the temperature range for phase transition from solid to fluid (-10°C to 0°C) during the thawing phase is significantly greater at longer thawing phases (see -10°C isotherm depth and ice volume after first freezing and first thawing phase in Table 1). After the thawing period the second freezing phase is started, leading to an immediate drop of temperatures especially in the fully frozen volume. The depicted isotherms reach approximately the same depth at the end of the second freezing phase (see also Table 1). As shown in a work of Chua and Chou [14], the simulated volume being thawed during the first thawing phase and refrozen during the second freezing phase showed significantly lower viability compared to scenarios with shorter thawing periods, in which the corresponding volume remained in the frozen state. This can be explained by the increased influence of thawing effects during longer thawing periods and the repetition of the effects induced by freezing. During the interim thawing period with 5 s duration the phase change boundary of -10°C does not recede to the depth of the necrotic -20°C isoline reached during the first freezing phase, indicating a higher chance of cell survival in the myocardial range between 3 mm and 3.5 mm transmural depth (see Figure 6a). In the simulation with 10 s rewarming the isoline recedes to a distance closer to the applicator than the -20°C isoline in the first freezing phase, closing this gap and increasing the chance of lower viability in this transmural range.

Figure 6b depicts the ice (solidus temperature isotherm) volume progress over time in the myocardium showing similar characteristics. In the ablation protocols with 15 s and 20 s interim thawing duration, the whole ice volume created during the first freezing phase enters the temperature range of phase change again and is refrozen during the second freezing cycle. Despite the significant differences in the minimal volume during the first thawing phase, approximately equal ice volumes are reached at the end of the second freezing phase for all simulated protocols.

**Figure 7:**
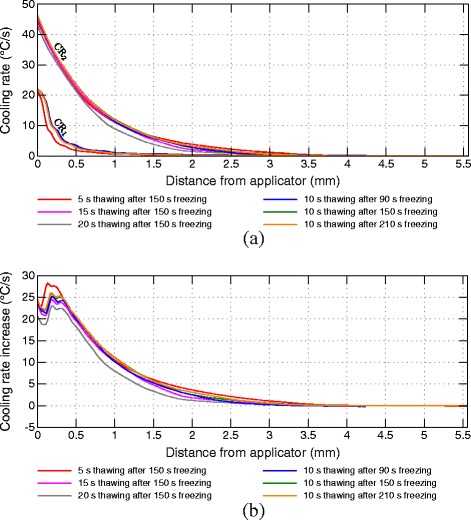
**Comparison of averaged cooling rates over transmural depth.** **(a)** Cooling rates of first (CR_1_) and second freezing phase (CR_2_). **(b)** Cooling rate increase in second freezing phase.

**Figure 8:**
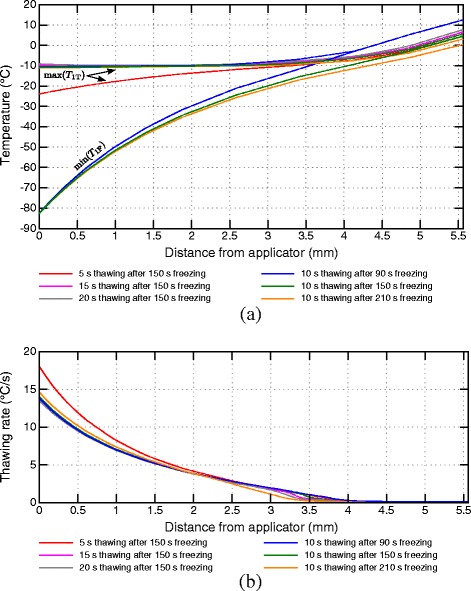
**Temperature ranges and thawing rates of the interim thawing phase.** Temperature ranges **(a)** and thawing rates **(b)** of the interim thawing phase over transmural depths for simulated ablation protocols. In **(a)** min(*T*_1F_) marks the lines of the minimum temperatures during the first freezing phase and max(*T*_1T_) marks the maximum temperatures during the interim thawing phase. The green line starting at -82.6°C defines the minimal temperature of the first freezing phase of all protocols with an initial freezing phase duration of 150 s.

In Figure 7, the results of the cooling rate comparison between the first and second freezing phases are summarized for different scenarios over the transmural depth. Figure 8 depicts the common temperature ranges of both freezing phases and thawing rates for different thawing phase durations and starting times. Higher temperature changes and thawing rates can be observed closer to the endocardium due to the more direct impact of the applicator and the fast rewarming of the tissue by the nearby blood stream. At farther distances the respective temperature ranges are reduced implying a smaller influence of the interim thawing phase. Due to the common start of the interim thawing phase, the lower boundary of the common temperature range is equal for the protocols with varying thawing durations. For the protocol with 5 s interim thawing phase the maximal thawing temperature close to the applicator is significantly lower compared to longer thawing durations. At longer thawing durations the tissue reaches temperatures close to -10°C. Only small variations of the maximum interim thawing temperature are visible for these protocols due to the latent heat considered by the effective heat capacity model in the phase change temperature range between -10°C and 0°C.

For shorter thawing durations the increase of the cooling rate depicted in Figure 7b is higher compared to longer thawing durations, especially at distances close to the applicator. More myocardial volume undergoes phase change again at longer thawing durations leading to a higher heat capacity at the beginning of the second freezing phase by latent heat resulting in lower cooling rates compared to shorter interim thawing phases.

The thawing rates depicted in Figure 8b show a similar characteristic. Although the thawing rates are equal at the beginning of the interim thawing phases after 150 s freezing, different temperatures are reached when the second freezing phase is started. The highest thawing rate was calculated for the shortest interim thawing phase of 5 s. During the short temperature rise in this protocol the lower phase change boundary is not reached. The crossing of the phase change boundary in simulations with longer thawing durations leads to a reduced thawing rate in these protocols.

### Initial freezing duration

The volume of myocardial tissue affected by the interim thawing phase depends on the duration of the initial freezing phase. The duration defines the size of the iceball, in which the effects of recrystallization take place, and furthermore the extent of the precooling of surrounding tissue, which affects the cooling rate of the second freezing phase.

**Figure 9:**
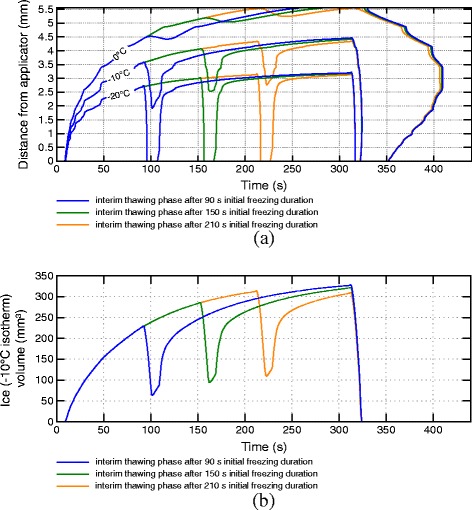
**Isotherms of transmural temperatures and ice volumes for varying initial freezing phase durations.** Isotherms of transmural temperatures **(a)** and iceball volumes **(b)** over time for ablation protocols with 10 s thawing phase starting after different initial freezing durations and a cumulated freezing duration of 300 s.

Figure 9 shows the isotherms of the transmural temperature profile and ice volume over time for the ablation protocols with varying starting times of the interim thawing phase (scenarios D1-D3 in Figure 4). The depth and volume crossing the solidus temperature of -10°C twice during the first thawing and second freezing phase varies depending on the start of the first thawing phase. A quantitative summary of transmural depths of the shown isotherms and the ice volume is given in Table 2. Beyond longer initial freezing phases the recrystallization and osmotic effects of the interim thawing phase are extended to areas more distant from the applicator and, consequently, a greater volume is affected. Variations in the starting time of the interim thawing phase have a minimal influence on the depths of the -10°C and -20°C isotherms after the second freezing phase. A later start of the interim thawing phase leads to slightly warmer temperatures at the end of the second freeze, mainly caused by the shorter second freezing cycle. The depicted results of the ablation protocol starting the interim thawing phase after 210 s also show that the ice volume of the first freeze is not reached completely after the second freezing phase. Also, a greater volume is rewarmed to temperatures above the lower phase change boundary and is being refrozen in the second cycle at later starts of the interim thawing phase.

**Table 2:** **Transmural isotherm depths and ice volumes for varying initial freezing phase durations**

**Thaw start**	**1F**			**1T**			**2F**		
**(s)**	**-10°C**	**-20°C**	**IV**	**-10°C**	**-20°C**	**IV**	**-10°C**	**-20°C**	**IV**
	**(mm)**	**(mm)**	**(mm** ^**3**^ **)**	**(mm)**	**(mm)**	**(mm** ^**3**^ **)**	**(mm)**	**(mm)**	**(mm** ^**3**^ **)**
90	3.6	2.7	230	1.9	0.0	64	4.5	3.2	328
150	4.1	3.0	286	2.5	0.0	95	4.4	3.2	322
210	4.3	3.1	314	2.8	0.0	110	4.3	3.1	310

Transmural depths of selected isotherms and ice (tissue below solidus temperature of -10°C) volumes (IV) for ablation protocols with varying starting times of interim thawing phase of 10 s and a cumulated freezing duration of 300 s.

In Figure 7 the cooling rates of the first and second freezing phases are compared for different starting times of the interim thawing phase. Figure 8a depicts the difference of the temperature ranges for different thawing phase start times. Warmer minimal temperatures at the end of the shorter first freezing phase consequently lead to smaller temperature ranges for rewarming and recooling, especially at farther distances from the applicator. The varying estimated cooling rates are caused by different precooling conditions of the surrounding tissue as well as by the variations of temperature ranges of rewarming and refreezing during the interim thawing and second freezing phase. Shorter initial freezing phases lead to a reduced increase of the cooling rate during the second freezing phase, especially for farther distances from the applicator. Small enhancements of the cooling rates are identifiable in protocols with a longer initial freezing phase in transmural depths of 4 mm, whereas no increase was measured for shorter initial freezing phases.

## Discussion

The treatment of cardiac arrhythmias by cryoablation is gradually gaining more importance [21] and, therefore, a better understanding of the processes in the treated myocardial tissue is essential to optimize both the procedure itself and the outcome. Although multiple in-vitro and in-vivo studies are available investigating the effects of CCA (e.g. [7,17,20,22,23]), only a few studies employ in-silico models for a closer investigation of temperature distributions over time in the field of CCA, including the studies published by our group [9-11]. Using in-silico models various procedures can be simulated and interpreted without shortcomings due to measurement biases induced by the instruments used or by anatomical variations. Additionally, a more detailed investigation of temperature fields is possible using computer models, allowing the interpretation of results, which experimentally can, if at all, only be measured with much higher efforts. Furthermore, ethical aspects have to be considered in in-vivo studies, whereas various conditions can be tested using in-silico models without the need and danger of harming subjects.

To analyze transmural temperatures for the evaluation of different ablation procedures we verified our model using temperature recordings from literature. In a study by Wood et al. [7] myocardial sections of porcine hearts were ablated using different tip applicators, among those also a 9 Fr 8 mm tip applicator was used. As the transmural temperature recordings of ablations using superfusate flow were biased due to conductive warming of the array holding the thermal probes as stated by the authors, measurements without superfusate flow were used for comparison with a slightly modified version of our model to consider thermally inactive tissue (deactivation of perfusion and metabolic heat contribution) and a steady blood layer above the applicator (deactivation of perfusion in the blood layer and adaptation of the surrounding boundary conditions). A good approximation to the measurements of Wood et al. was found (see Appendix), verifying simulations of our model also in regions more distant from the cryoprobe. However, measuring transmural temperatures during cryoablation in-vivo remains a challenging task. Promising approaches have been developed for the recording of temperatures in-situ by Thaokar & Rabin [24] allowing for a more accurate insight into temperature distributions during thermal ablation procedures.

To investigate only the effects of different modalities concerning multiple freeze-thaw cycles the overall freezing duration of the simulated ablation procedures compared in this study was set to 300 s. Longer overall freezing durations were not simulated due to the quasi-static state of the temperature field at the end of the continuous 300 s freezing phase (maximal temperature change approximately -0.02°C s ^−1^ after 300 s freezing) implying only small variations in transmural depth and volume of relevant isotherms. Furthermore, shorter ablation scenarios lead to a higher clinical acceptance. Nevertheless, it is known from literature that longer freezing durations enlarge the ablated tissue region, especially in the temperature range between -10°C and -25°C due to increased solute effects and recrystallization [3,6].

The analysis of different thawing durations after a freezing phase of 150 s shows that the temperature distribution within the myocardium is strongly influenced by short thawing phases of only a few seconds. The fast rewarming of both the tissue and the applicator can lead to a disengagement from the endocardium and the applicator may be dislocated in the second freezing phase, if longer thawing durations are chosen. Our simulations show that already after 90 s freezing the thawing duration until the iced applicator tip reaches the lower phase change boundary is only marginally dependent on the initial freezing duration. Although effects of recrystallization and solute effects are smaller at faster rewarming rates of previously frozen tissue over short periods of time [6,12,14,25], an interim thawing phase enhances the cooling rate of the second freeze-thaw cycle, thus increasing the chance of intracellular ice formation. The increase of the cooling rate depends both on the distance to the applicator and the thermal history of the tissue. While shorter thawing periods lead to a higher increase of the cooling rate in the second freezing phase compared to longer thawing periods, the higher cooling rate is only effective in smaller temperature ranges compared to longer thawing phases. Additionally, longer thawing periods result in a greater volume to undergo phase change again and being refrozen during the second freezing phase. The thawing duration should, therefore, be chosen carefully to maximize the range being influenced by the thawing phase while keeping the applicator adhered to the initial ablation site. Thawing durations can be prolonged if a close positioning of the applicator to the first ablation location can be guaranteed, for instance by the usage of specialized navigation systems.

The region influenced by multiple freeze-thaw cycles also varies according to the duration of the initial freezing phase. While shorter initial freezing durations cause regions closer to the applicator to undergo phase change again during the interim thawing phase, longer initial freezing phases shift the effect to regions more distal to the applicator. Additionally, the affected volume is greater when the interim thawing period is started later in the scenario. This finding indicates that adaptations of the interim thawing phase starting time are an important factor for the optimization of ablation protocols depending on the myocardial wall thickness of the relevant ablation site.

Characteristic phases of the refrigerant nitrous oxide (N _2_O) were considered in the simulations as previously described in [10]: At the beginning of a freezing phase, the cooling power of the applicator continuously increases during the filling of the supply lines and the raise of the refrigerant flow rate. This consequently influences the cooling rates at the beginning of the freezing phases. At the end of a freezing phase the valve in the supply line is closed. The rewarming of the applicator is slightly delayed until the remaining refrigerant in the applicator is completely drained. By considering the characteristic refrigerant phases, a realistic cooling and rewarming behavior of the applicator and the tissue was simulated for the discussed ablation scenarios.

In our work, averaged material properties (thermal conductivity, specific heat capacity, blood perfusion rate and metabolic heat generation rate) obtained from literature were used in combination with a simplified geometrical model [10]. Although temperature distributions are affected by variations of material properties and geometries, the general trends demonstrated in our simulations remain unchanged. It can be expected that large blood vessels have an influence on transmural temperature distributions during cryoablation and, therefore, on the ablation result. The examination of effects caused by anatomical variations was not considered in the present model. Our results are based on averaged conditions and allow for drawing reasonable conclusions on specific ablation protocols.

Transmural temperature profiles were analyzed between the coldest point at the epicardium after 300 s freezing and the applicator tip, where the transmural extent of relevant isotherms (phase change boundaries, lethal temperature) is the largest. Along this transmural path, the effects of varying ablation scenarios can be examined best. In addition, the temperature distribution along this path can also be used for comparison with lesion depths from literature due to the fact that lesion depths are also measured by the maximal transmural extent of the lesion.

The presented simulations in this work provide better insight into temperature distributions over transmural depths during CCA, which may contribute to optimization strategies depending on the wall thickness of the treated myocardium and furthermore allow for an improvement of ablation protocols in CCA. In addition to increased ablation success, another improvement is the reduction of intervention time. Obviously, shorter freezing durations are reasonable for thinner myocardial wall thicknesses as lethal temperatures reach the epicardium in shorter time spans. By incorporating multiple freeze-thaw cycles, the ablation time can be decreased by additionally investigating the volumes undergoing phase change during interim thawing phases and being refrozen in the following freezing phase.

## Conclusions

In summary, we used a powerful simulation model, which was verified by in-vivo experiments confirming the temperature profiles at the applicator, and in-vitro results from literature for temperature profiles over transmural depth. This model allows for the analysis and optimization of established cryoablation scenarios and for the development of new ablation strategies for future CCA studies. This may contribute to a higher clinical acceptance for treating cardiac arrhythmias using this technology.

## Limitations

Simulated temperature distributions were solely verified by in-vivo temperature measurements at the applicator tip [10] and in-vitro temperature recordings of myocardial tissue as shown in [7] (see Appendix). In addition, iceball depths (depths of -10°C isotherms) were compared with lesion depths from literature [17,20].

A quantification of ablated tissue regions (lesion depth, lesion volume) was not possible due to a lack of experimental data for the simulated ablation protocols. For a comprehensive assessment of protocol parameters (such as interim thawing duration, thawing start time, and overall ablation duration) on ablation results in CCA, in-vivo experiments with a sufficient number of ablations are needed and are objectives of future studies.

## Appendix

### Verification of transmural temperature profiles

In the work of Wood et al. [7], sections of porcine myocardium (20×20×10 mm) were ablated using a tip applicator with equal dimensions (9 Fr diameter, 8 mm electrode length) analogously used in our model. Temperature profiles in the myocardium were recorded using optical temperature probes fixed by a small plastic frame and positioned 1 mm, 2 mm, 3 mm and 5 mm below the surface in contact with the applicator. Experiments were performed with and without superfusate flow over the electrode-tissue interface. The authors state in their study limitations that the array carrying the temperature probes was exposed to the superfusate and, consequently, the tissue and temperature probes were warmed by the superfusate during the ablation. Therefore, the measurements without superfusate flow were used for comparison with an adapted model.

The modified model contains an inactive tissue layer (*Q*_*m*_=*Q*_*p*_=0) with equal dimensions (20×20×10 mm), an inactive blood layer (*Q*_*p*_=0) of 3 mm thickness and adapted boundary conditions at the blood and tissue boundaries *a* and *b* (the heat transfer coefficient *α* was reduced from 1500 W m ^−2^ °C ^−1^ to 700 W m ^−2^ °C ^−1^ to consider water at rest and the external temperature *T*_*c*_ was set to 37°C).

**Figure 10:**
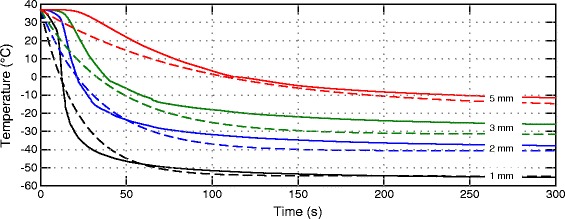
**Comparison of transmural temperature profiles in-silico vs. in-vitro.** Comparison of transmural temperature profiles obtained from simulation (solid lines) and from Wood et al. [7] (dashed lines) in 1 mm, 2 mm, 3 mm and 5 mm below the applicator-tissue contact area.

Temperatures were extracted 1 mm, 2 mm, 3 mm and 5 mm beneath the applicator and compared with the measurements of Wood et al. [7] simplified as fitted monoexponential functions in their work (see Figure 10). Transmural temperatures in-vitro [7] vs. in-silico after 300 s freezing are in a similar range (see Figure 10 and Table 3).

**Table 3:** **Transmural temperatures in-vitro [** 7 **] vs. in-silico after 300 s freezing**

**Distance (mm)**	**In-vitro (°C) [** 7 **]**	**In-silico (°C)**
1	-54.5 ± 4.1	-55.3
2	-40.7 ± 7.2	-37.9
3	-31.5 ± 7.0	-26.1
5	-16.8 ± 4.9	-11.5
